# Abdominal Dropsy Complicating Pregnancy

**Published:** 1882-01

**Authors:** E. T. Haygard


### VI.—ABDOMINAL DROPSY COMPLICATING PREGNANCY.

REPORTED BY E. T. HAYGARD, V.S.

Visited short-horn cow belonging to Mr. G. Whitney. She was supposed to be about a month from calving. In fair condition; abdomen much distended; some œdema of hind legs; considerable difficulty in breathing, especially when lying down, and increased materially during the heat of the day; micturition irregular and in small quantities, but general health good.

The indications were uterine or abdominal dropsy, but as the period of parturition was approaching, it was not considered advisable to submit the cow to an examination, vaginal or rectal, until after that time. In the meantime diuretics were prescribed and directions given in regard to diet, etc.

On the 26th Mr. Whitney reported the cow worse. On visiting her it was seen the distension had materially increased and the breathing had become so difficult that suffocation was imminent. There was no preparation for calving, no springing of the udder, no swelling of the vulva or relaxation of the pelvic ligaments. On examination by the rectum the uterus could be distinctly felt, and in it a rounded irregular mass, which to the feel had the appearance of a tumor or a fœtus arrested in its development and enveloped in its membranes. The uterus being so distinctly felt, and its only contents apparently being the mass described—although the whole appeared to be floating in fluid—together with the evident fluctuation of fluid in the abdominal cavity, lead me to the conclusion that the effusion was limited to the peritoneal sac. Paracentesis was determined upon, the puncture being made on the right side, in front of the udder, and about five or six inches to the right of the umbilicus. It was calculated that not less than forty-three gallons of serum were drawn off. The cow bore the operation well, and a few minutes after the administration of a stimulant appeared quite ready for her usual allowance of food.

The day after the operation a dead fœtus was expelled. I did not see it, but it was reported to me as corresponding in form and description to the mass I felt in the uterus before the operation. Alternate doses of tonic and diuretic medicine were prescribed, and the cow is now reported to me as quite well.

LEXINGTON, KY., October 9, 1881.

[The outlines of this case would lead one to infer that it was one of ascites due to portal obstruction. As the urine was not examined, it is impossible to draw any positive conclusion regarding renal causation. Albumenuria is quite common during pregnancy in human subjects, and may be in lower animals. Careful analysis of the urine, however, has not had the attention which it deserves in comparative medicine, and until it does, many diseases must pass undiagnosticated.

The diagnosis in this case seems to lie between intro-uterine dropsy and abdominal dropsy, probably the latter.

This case might be classed under the head of a missed-labor with the secondary accumulation of fluid in the abdominal cavity.—ED.]

